# Production of Defense Phenolics in Tomato Leaves of Different Age

**DOI:** 10.3390/molecules25214952

**Published:** 2020-10-26

**Authors:** Kateřina Dadáková, Tereza Heinrichová, Jan Lochman, Tomáš Kašparovský

**Affiliations:** Department of Biochemistry, Faculty of Science, Masaryk University, Kotlářská 2, 61137 Brno, Czech Republic; 474184@mail.muni.cz (T.H.); jlochman@seznam.cz (J.L.); tkasp@sci.muni.cz (T.K.)

**Keywords:** tomato, phenolics, defense compounds, bacterial infection, plant age, leaf age

## Abstract

Phenolics play an essential role in the defense reaction of crop plants against pathogens. However, the intensity of their production induced by infection may differ during the life of a plant. Here, we identified age-related differences in phenolic biosynthesis in the pathosystem *Solanum lycopersicum* cv. Amateur and *Pseudomonas syringae* pv. tomato DC3000. We analyzed concentrations of total phenolics, phenolic profiles, and concentrations of selected phenolic acids. The influence of bacterial infection, together with leaf and plant age, was assessed. The changes in concentrations of caffeic acid, 4-hydroxybenzoic acid, and salicylic acid glucoside caused by infection were found to be influenced by age. In concrete, the increases in the concentrations of these metabolites were all evident only in young plants.

## 1. Introduction

Plants are exposed to a range of pathogenic organisms, including bacteria, fungi, and viruses, resulting in infections and substantial reductions in crop yields. Modern methods of crop protection often exploit the natural plant defense reaction against pathogens, allowing for reducing the amounts of pesticides contained in foodstuffs and released into the environment [1,2]. Plant defense-related metabolites accumulate due to defense stimulation or infection and influence the quality of harvested plant products [3]. Therefore, studying the defense reaction and defense-related secondary metabolites of crop plants is of great importance.

Among the defense-related secondary metabolites, phenolics play an essential role throughout the plant kingdom. Due to their chemical structure, they act primarily as antioxidants and accumulate in the tissues, especially in the cases of microbial infections connected with oxidative stress [4]. Following reaction with a radical during oxidative stress, hydroxyl groups attached to aromatic rings enable phenolic compounds to be converted to stable radical forms, terminating the production of new reactive species [5]. Furthermore, phenolic compounds play important roles in repelling pathogens and pests, and many phenolics possess direct antimicrobial properties [6].

The concentrations of phenolics in plant tissues are known to increase in response to infections by microbial pathogens [6]. Phenylalanine ammonia-lyase, a key enzyme of phenolic synthesis, is commonly induced during the so-called pattern-triggered immunity, which is activated after the recognition of a microbe-associated molecular pattern [7]. Apart from this basal resistance, phenolics are also involved in specific interactions between host plants and their pathogens, including tomato defense against bacterium *Pseudomonas syringae* pv. tomato DC3000. In resistant tomato cultivars, recognition of the *P. syringae* bacterium leads to induction of the phenylpropanoid pathway and this way to cell wall lignification and thickening, as well as to enhanced synthesis of anthocyanins and other antioxidants [8].

However, the content of phenolics in plants is dynamic and changes according to environmental and developmental conditions [6,9]. In crop plants, phenolics content in leaves somewhat decreases with age, likely because of the decrease in photosynthate supply during leaf maturation [10,11,12,13]. This trend was generally confirmed in tomato, but different phenolic compounds followed different patterns during leaf development and aging [9].

The aim of this study was to identify age-related differences in the phenolic biosynthesis in the well-described model pathosystem *Solanum lycopersicum* cv. Amateur and *Pseudomonas syringae* pv. tomato DC3000. For the first time, we have summarized the age-related differences in the biosynthesis of defense phenolics in tomato and described the identified changes in the contents of phenolic acids.

## 2. Results and Discussion

### 2.1. Total Phenolic Contents

Total concentrations of phenolics determined by the reaction with Folin-Ciocalteu reagent ranged between 20 and 70 µg of gallic acid equivalents (GAE) per 1 mL of leaf extract (0.8–2.8 mg GAE/g of leaf DW, Table 1). Generally, the mean concentrations of phenolics were lower in control than in infected leaves, but these differences were not statistically significant (*p* > 0.2). This observation could be explained by simultaneous production of phenolics and their transformation into more antimicrobial compounds during defense reaction, as suggested in an earlier study on tomato leaves [14]. In previous studies, the leaf extracts from tomato plants displayed contents between 6 and 130 mg GAE/g of the dried extract [15,16] or 160–240 mg GAE/g of leaf DW [17]. The wide range of phenolic concentration values found in leaves is likely caused by natural variations connected to the different genetic background as well as to the developmental and environmental conditions of studied plants.

### 2.2. Phenolic Profiles

**Figure 1:**
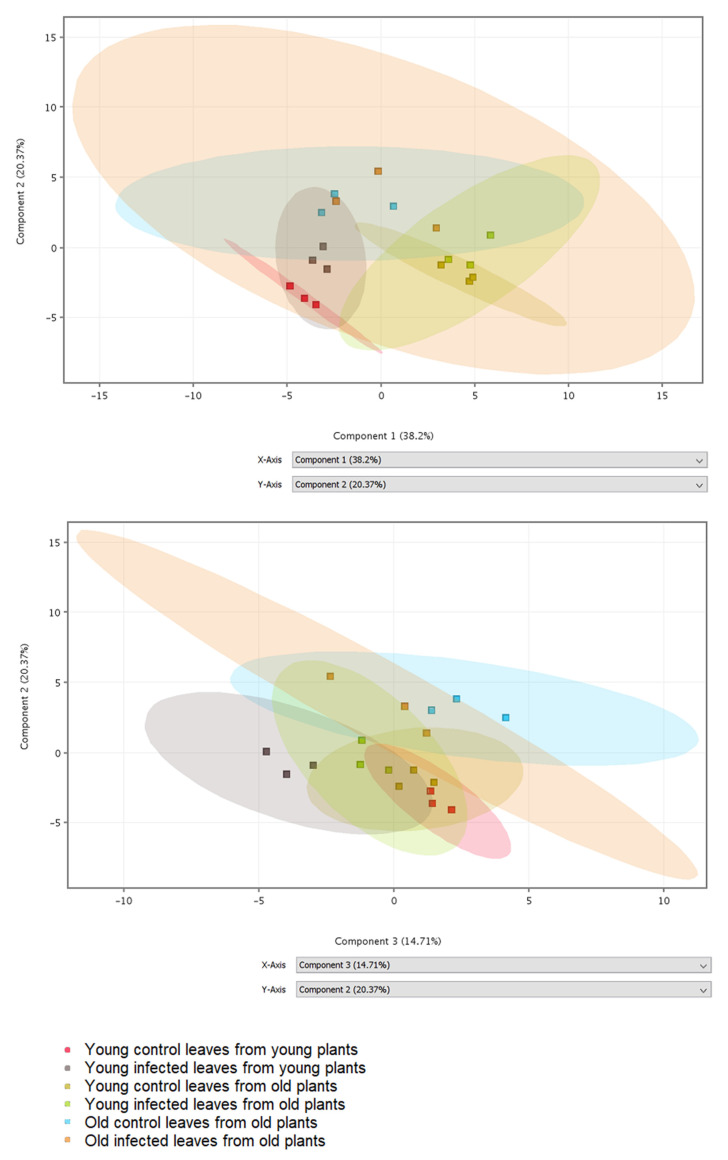
2D visualization of principal component analysis (PCA) using the first three components. Different samples (three biological replicates per variant) are shown in different colors.

Phenolic compounds and compounds from biosynthetic pathways of phenolics were detected in leaf extracts by LC-MS. In total, 37 metabolites were putatively identified by comparison of obtained mass spectra with database records (Table S1). Differences in the obtained phenolic profiles were visualized by principal component analysis (PCA) (Figure 1). The 2D visualization shows that component 1 separates the samples according to the plant age, component 2 separates the samples according to the leaf age, and component 3 separates control and infected samples, primarily in young plants. The PCA loadings (Table S1) indicate that component 1 includes primarily compounds putatively identified as benzoic, syringic, 4-hydroxybenzoic, and ellagic acids, all with negative coefficients; their amounts seem to decrease with increasing plant age (Figure 1). Component 2 includes primarily compounds putatively identified as kaempferol derivatives, again with negative coefficients. The last component 3 includes compounds putatively identified as ferulic and chlorogenic acids with negative coefficients and flavonoid derivatives rutin and kaempferol glycoside with positive coefficients; their amounts seem to decrease with infection.

### 2.3. Concentrations of Selected Phenolic Acids

**Figure 2:**
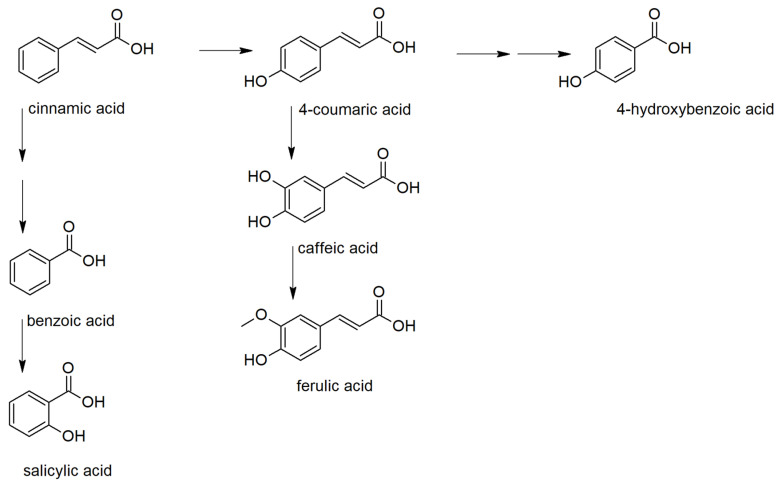
Biosynthetic relations between the studied phenolic acids.

Based on the PCA results, phenolic acids, their precursor, and derivatives were analyzed in detail (Figure S1). Phenolic acids serve as precursors for many important metabolites influencing the plant fitness. They can be classified as derivatives of benzoic and cinnamic acids (Figure 2) [18]. Among benzoic acid derivatives, the plant hormone salicylic acid is probably the most important metabolite concerning the defense reaction against pathogens. In plants, it is synthesized either from chorismate by isochorismate synthase (ICS) pathway or by phenylalanine ammonia-lyase (PAL) pathway, including benzoic acid 2-hydroxylase [19,20]. The importance of these pathways for the biosynthesis of salicylic acid differs in plant species.

**Figure 3:**
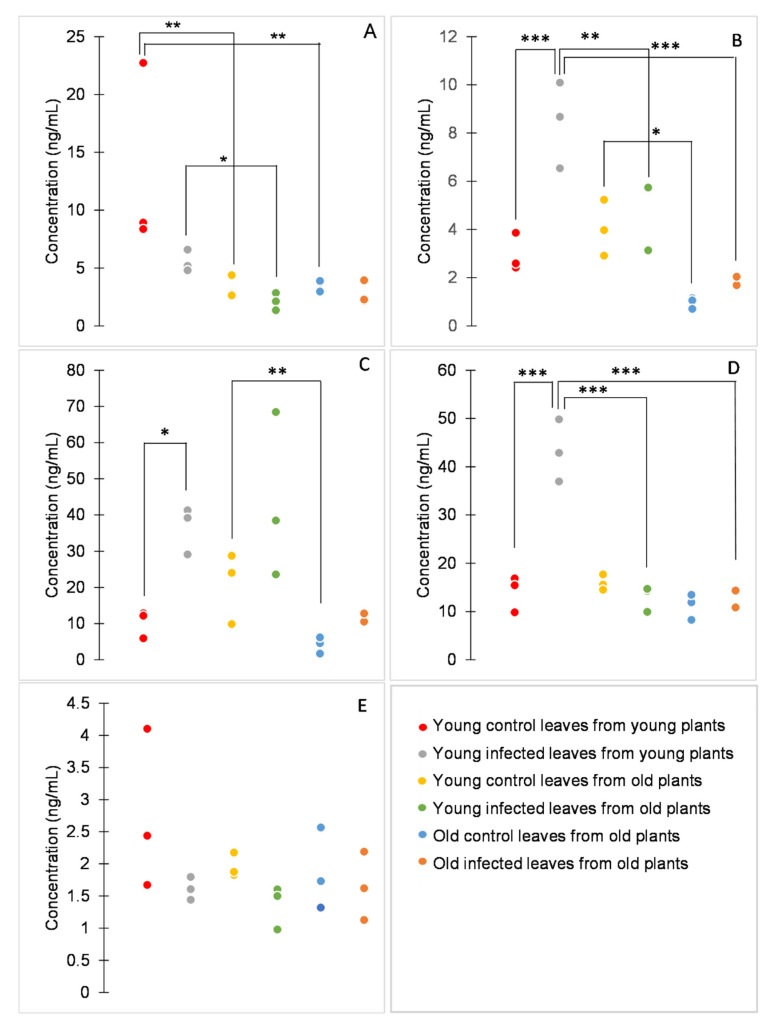
Concentrations of benzoic (**A**), caffeic (**B**), ferulic (**C**), 4-hydroxybenzoic (**D**), and syringic acid (**E**). Dot plots display all data points (three biological replicates per variant). *p* values were determined using factorial ANOVA, * indicates *p* ≤ 0.05, ** indicates *p* ≤ 0.01, *** indicates *p* ≤ 0.001. For better clarity, only statistically significant differences between samples of either the same age or the same infection status are shown.

The comparison of retention times with those of analytical standards showed that chlorogenic and ellagic acids had been misidentified. Benzoic, caffeic, ferulic, 4-hydroxybenzoic, and syringic acid were correctly identified and quantified (Figure 3).

Among the analytes, the concentration of syringic acid was significantly influenced neither by age nor by *P. syringae* infection (Figure 3E). Other analytes were influenced by both age (*p* < 0.001 for benzoic, caffeic, hydroxybenzoic, and ferulic acids) and infection (*p* = 0.011, *p* = 0.002, *p* < 0.001, and *p* < 0.001 for benzoic, caffeic, 4-hydroxybenzoic, and ferulic acid, respectively), or also by their interaction (*p* = 0.002 and *p* < 0.001 for caffeic and 4-hydroxybenzoic acid, respectively; Figure 3A–D).

According to the age effect, the studied phenolic acids can be divided depending on whether there is a difference between plants of different ages or between leaves of different ages. The concentration of benzoic acid was significantly higher in young healthy plants than in old healthy plants and also in young infected plants than in young leaves of old infected plants. The concentration of 4-hydroxybenzoic acid was significantly higher in young infected plants than in old infected plants. On the other hand, the concentration of ferulic acid was significantly higher in young healthy leaves than in old healthy leaves of old plants. The concentration of caffeic acid was significantly higher in young healthy leaves from old plants than in old healthy leaves from old plants. Furthermore, the concentration of caffeic acid was significantly higher in infected young leaves from young plants than in both types of infected leaves from old plants (Figure 3). In a previous study on tomato leaves, two phenolic compounds were putatively identified, caffeic acid glucoside and rutin [9]. Caffeic acid glucoside showed a decrease in content with increasing leaf age, in concordance with the result reported here. Moreover, in this study, the content of other phenolic acids was found to decrease with the age of the tissue.

Concerning the infection effect, the concentrations of caffeic, ferulic, and 4-hydroxybenzoic acids tended to be higher in infected than in healthy tissues, concordant with previously reported results [21,22,23]. However, the effect was evident only in young plants. Ferulic acid and its precursor caffeic acid have been studied for their antimicrobial properties [24,25]. Moreover, increased concentration of caffeic acid has been associated with tomato response to bacterial pathogens and with enhanced resistance against *P. syringae* [26,27]. Therefore, it is not surprising that these phenolic acids are synthesized in tomato leaves in response to this pathogen.

Furthermore, a compound putatively identified as salicylic acid glucoside was significantly influenced by infection, age, and their interaction (*p* = 0.020, *p* < 0.001, and *p* < 0.001, respectively). In concrete, the amount of this compound was significantly higher in young infected leaves of young plants than in all other samples (*p* < 0.01, Table 2). This observation is similar to that of Scalschi et al., who found increased amounts of salicylic acid in tomato 48 h after infection with *P. syringae* [28].

## 3. Materials and Methods

### 3.1. Chemicals, Plant, and Fungal Material Inoculation

Phenolic acid standards were purchased from Sigma-Aldrich (caffeic, chlorogenic, ferulic, 4-hydroxybenzoic, and syringic acid, Darmstadt, Germany), Penta (benzoic acid, Prague, Czech Republic), and Cayman Chemicals (ellagic acid, Michigan, MI, USA).

Tomato plants (*Solanum lycopersicum* cv. Amateur) were grown in 25 °C, 50% humidity and 16 h light period. Three types of leaves were inoculated with *Pseudomonas syringae* pv. tomato DC3000 (kindly provided by prof. Galaud, Université Paul Sabatier (Toulouse, France): young leaves (first and second fully developed from the top of the plant) from young plants (6.5 weeks old), young leaves from old plants (12 weeks old), and old leaves (fourth and fifth fully developed) from old plants. In total, three young and three old plants were used: a mixture of two leaves from one plant was considered a biological replicate. Inoculation was done by immersing the detached leaves into *P. syringae* suspension (10^7^ bacteria per mL) with 0.0025% surfactant (Silwet, General Electric Company, Fairfield, Connecticut, CT, USA). Control leaves were immersed into 0.0025% surfactant in water. Leaves were then kept in a wet place for two days, until they showed first disease symptoms. Afterwards, stems were removed, leaves from the same plant were mixed, and samples were kept at −70 °C until analysis.

### 3.2. Extraction and Analysis of Total Phenolic Contents

Leaves were dried at 60 °C. 50 mg of dry leaves were extracted twice with 1 mL 80% methanol by 1 min of vigorous mixing, 15 min of sonication, and 30 min of incubation (750 rpm, 25 °C). The total content of phenolics was assessed using Folin-Ciocalteu reagent, which oxidizes the hydroxyl groups of phenols. Briefly, 50 µL of Folin-Ciocalteu reagent was added to 100 µL of the extract, and after three minutes, 100 µL of sodium carbonate solution (5 g/L) and 750 µL of water were added. The mixture was incubated at 50 °C for 16 min. Then, absorbance was measured at 765 nm and compared with that obtained from the blank (without Folin-Ciocalteu reagent). The calibration curve was prepared using gallic acid. The results obtained were thus expressed as µg of gallic acid equivalents per 1 mL of leaf extract, i.e., 25 mg of dry leaves. Three biological replicates per condition were analyzed.

### 3.3. Analysis of Phenolic Profiles and Quantification of Phenolic Acids

1 mL of the leaf extract was heated to 50 °C, dried in a nitrogen stream, and dissolved in 1 mL of 5% methanol in acidic water (0.01% acetic acid). Samples were filtered and analyzed by HPLC-MS (6545, Agilent, Santa Clara, CA, USA). Analytes were separated using a reverse-phase column (EclipsePlus C18, 2.1 × 50 mm^2^, 1.8 µm, Agilent, Santa Clara, CA, USA) and a gradient elution. The mobile phases used were (A) 0.01% acetic acid and (B) methanol. The gradient conditions were as follows: 5 to 20% B from 0 to 10 min, 20 to 100% B from 10 to 20 min, and finally, 100% from 20 to 30 min, at a flow rate of 0.3 mL/min and injection volume of 5 µL. Electrospray ion source was used under the following conditions: acquisition mode 100–1700 *m*/*z*, gas temperature 300 °C, gas flow 8 L/min, ion polarity negative, capillary voltage 4000 V, fragmentor voltage 150 V.

Phenolic compounds and compounds from biosynthetic pathways of phenolics were detected by TOF and putatively identified by comparing obtained mass spectra with the database (Metlin, The Scripps Research Institute, La Jolla, CA, USA, metlin.scripps.edu). Selected phenolic acids were identified by comparison of retention times and mass spectra with those of respective standards. Successfully identified analytes were quantified using peak areas of [M − H]^−^ ions (Table 3). Calibration curves were drawn using standards in MassHunter Quantitative Analysis software (Agilent, Santa Clara, CA, USA https://www.agilent.com/en/products/software-informatics/masshunter-suite/masshunter-quantitative-analysis). Three biological replicates per condition were analyzed.

### 3.4. Statistical Analysis

Differences in total phenolic contents, concentrations of phenolic acids and peak areas for compound putatively identified as a salicylic acid glucoside were assessed using factorial ANOVA (the factors being infection and age). Due to unequal variances, concentrations of total phenolics, benzoic and ferulic acids were log-transformed before analysis. Tukey HSD test was used as a post-hoc test. The *p*-value cutoff for significant results was set to 0.05. Differences in phenolic profiles were visualized using principal component analysis (PCA) in Mass Profiler Professional 15.0 software (Agilent, Santa Clara, CA, USA, https://www.agilent.com/en/product/software-informatics/mass-spectrometry-software/data-analysis/mass-profiler-professional-software).

## 4. Conclusions

In young plants, the bacterial infection seems to cause the enhanced synthesis of salicylic acid glucoside from pre-formed benzoic acid. In old plants, the concentration of pre-formed benzoic acid is low; therefore, in the case of infection, no salicylic acid glucoside is synthesized. The data suggest that in the case of infection, benzoic acid is not newly produced, as carbon is redirected from the benzoic acid biosynthetic pathway through coumaric acid to antimicrobial caffeic, ferulic, and 4-hydroxybenzoic acids.

In older plants, differences can be seen between young and old leaves. Young leaves contain more ferulic acid and its precursor caffeic acid; therefore, they are supposedly better protected against bacterial infections than old leaves. However, the differences in the contents of phenolic acids, caused by a bacterial infection, are more subtle and statistically insignificant in old plants. Altogether, the data indicate that the responsiveness of several tomato phenolics to bacterial infection decreases with the plant age.

## Figures and Tables

**Table 1:** Total concentrations of phenolics expressed as gallic acid equivalents. Data are presented as means ± standard deviations of three biological replicates. Statistical significance was determined using factorial ANOVA, the same letter (a) indicates no significant differences.

Total Phenolics (µg/mL)	Young Plants	Old Plants	
	Young Leaves	Young Leaves	Old Leaves
Control	27 ± 6 (a)	20 ± 6 (a)	44 ± 14 (a)
Infected	48 ± 4 (a)	35 ± 8 (a)	70 ± 15 (a)

**Table 2:** Peak areas of extracted ion chromatograms (EIC) for compound putatively identified as a salicylic acid glucoside. Two *m*/*z* values (299.0772 and 359.0984) were merged into one chromatogram. Data are presented as means ± standard deviations of three biological replicates. Statistical significance was determined using factorial ANOVA, different letters indicate significantly different results.

Peak Area (kCounts × s)	Young Plants	Old Plants	
	Young Leaves	Young Leaves	Old Leaves
Control	687 ± 76 (a)	1372 ± 26 (a)	1090 ± 49 (a)
Infected	3661 ± 123 (b)	1867 ± 89 (a)	1438 ± 44 (a)

**Table 3:** *m*/*z* ratios of the successfully identified analytes.

Analyte	[M − H]^−^ (*m*/*z*)
Benzoic acid	121.0295
Caffeic acid	179.0350
Ferulic acid 4-hydroxybenzoic acid	193.0506
	137.0244
Syringic acid	197.0455

## Supplementary Materials

The following are available online. Table S1: List of putatively identified compounds. Figure S1: Mass spectra of both correctly identified and misidentified phenolic acids.
