# Supplementary material for: Production of Defense Phenolics in Tomato Leaves of Different Age
Source: Molecules. 2020 Oct 26;25(21):4952. doi: 10.3390/molecules25214952 (PMC7663536; doi:10.3390/molecules25214952)
Supplement: Supplementary file 1 [file molecules-25-04952-s001.pdf]

Supplementary Materials

Production of Defense Phenolics in Tomato Leaves of Different Age

Kateřina Dadáková *, Tereza Heinrichová, Jan Lochman and Tomáš Kašparovský

Department of Biochemistry, Faculty of Science, Masaryk University, Kotlářská 2, 61137 Brno, Czech Republic; 474184@mail.muni.cz (T.H.); jlochman@seznam.cz (J.L.); tkasp@sci.muni.cz (T.K.)

* Correspondence: 147047@mail.muni.cz

Academic Editors: Sławomir Dresler and Barbara Hawrylak-Nowak

Received: 25 September 2020; Accepted: 26 October 2020; Published: 26 October 2020

Table S1: List of putatively identified compounds.

**Putative Identity**	**Mass (avg)**	**RSD (Mass, ppm)**	**Retention Time (min)**	**PCA Component Loadings**		
				Comp. 1	Comp. 2	Comp. 3
Flavonoids and flavonoid derivatives						
dihydrokaempferol	288.0631	1.33	17.5	-0.160	-0.278	0.009
2,4',5,7-tetrahydroxyisoflavanone	288.0631	1.35	17.5	-0.161	-0.277	0.012
wighteone	338.1153	1.01	17.6	-0.108	-0.157	0.131
luteone	354.1116	5.79	18.0	-0.023	-0.028	0.243
epigallocatechin	306.074	1.49	8.9	-0.245	-0.093	0.074
kaempferol-3-glucoside-7-rhamnoside	594.1595	0.96	18.0	0.075	-0.284	0.194
rutin	610.1541	0.55	17.5	-0.027	0.070	0.324
kaempferol-3-gentiobioside	610.1534	0.55	17.5	-0.027	0.070	0.324
quercetin-3-glucoside	464.0957	0.59	17.5	0.191	-0.238	-0.013
kaempferol-3-glucoside	448.1013	0.95	18.1	0.116	-0.317	-0.028
quercetin 3-(2G-xylosylrutinoside)	742.197	0.6	16.9	0.187	-0.163	0.141
kaempferol 3-[2''-(6'''-coumaroylglucosyl)-rhamnoside] 7-glucoside	902.2531	3.83	18.0	0.213	-0.075	0.186
kaempferol 3-(2-p-coumaroylsophoroside) 7-glucoside	918.2452	1.58	17.7	0.236	-0.050	0.135
delphinidin derivative	465.0998	2.24	17.5	0.184	-0.248	-0.003
Chalcone						
isoliquiritigenin	256.0732	1.41	19.5	0.000	-0.321	-0.166
Metabolites involved in biosynthetic pathway of phenolics						
L-phenylalanine	165.0786	0.73	5.7	-0.228	-0.089	0.101

succinate	118.0264	0.95	3.5	-0.215	-0.000	-0.216
benzoic acid	122.0364	1.05	17.4	-0.237	-0.140	0.028
glucose	180.0629	0.95	1.5	-0.060	0.162	0.343
rutinose	326.1208	0.83	3.3	-0.008	0.088	-0.151
2-oxoglutarate	146.0209	0.81	3.3	-0.217	-0.029	-0.137
cinnamic acid	148.052	0.78	5.7	-0.204	-0.103	0.102
Phenolic acids and phenolic acid derivatives						
caffeic acid	180.0418	0.79	13.0	0.038	-0.182	-0.165
4-hydroxybenzoic acid	138.0314	0.84	12.7	-0.214	-0.086	-0.189
ferulic acid	194.0574	1.25	16.8	-0.041	-0.189	-0.296
4-coumarate	164.0469	0.81	12.6	0.013	-0.099	-0.039
vanillic acid	168.0419	1.05	14.7	-0.172	0.197	-0.159
veratric acid	182.0572	1	14.2	-0.128	0.211	0.098
syringic acid	198.0524	1.5	15.4	-0.237	-0.066	0.092
ellagic acid	302.0081	0.76	1.4	-0.256	-0.049	-0.008
5-O-caffeoylshikimic acid	336.0849	3.85	1.8	0.120	-0.041	-0.006
1-O-sinapoylglucose	386.1208	0.69	12.6	-0.213	-0.031	0.197
chlorogenic acid	354.0969	1.01	1.7	-0.053	-0.106	0.264
2-O-caffeoylglucarate	372.0674	2.78	14.5	0.177	-0.189	0.017
3-O-feruloylquinic acid	368.1105	0.91	15.1	0.210	0.045	-0.023
5-O-caffeoyl-D-quinate	353.0852	3.03	16.7	0.107	0.235	-0.162
O-feruloylgalactarate	386.085	0.73	14.4	0.188	0.092	-0.099

Figure S1: Mass spectra of both correctly identified and misidentified phenolic acids.

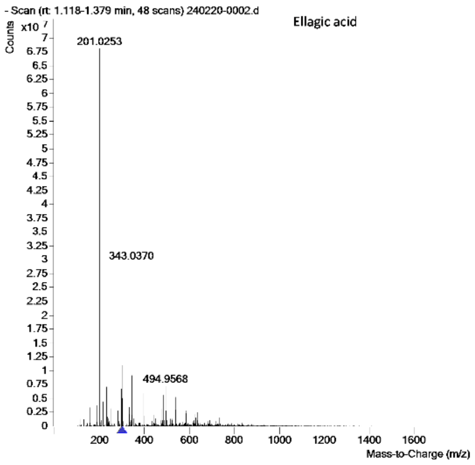

- Scan (rt: 1.118-1.379 min, 48 scans) 240220-0002.d
Ellagic acid
Counts
x10 7
201.0253
343.0370
494.9568
Mass-to-Charge (m/z)

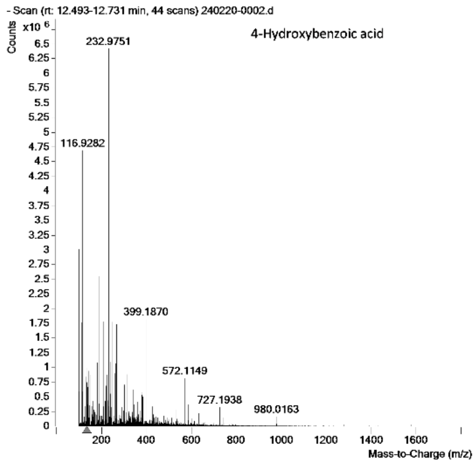

- Scan (rt: 12.493-12.731 min, 44 scans) 240220-0002.d
4-Hydroxybenzoic acid
Counts
x10 6
232.9751
116.9282
399.1870
572.1149
727.1938
980.0163
Mass-to-Charge (m/z)

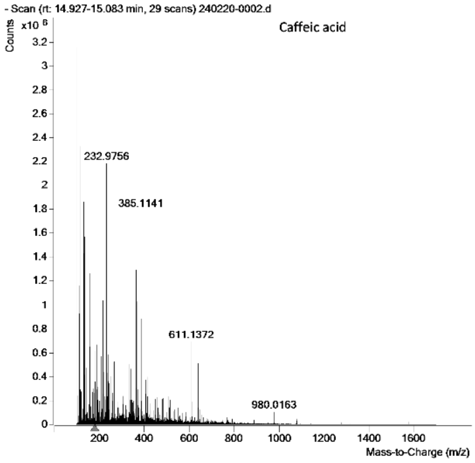

- Scan (rt: 14.927-15.083 min, 29 scans) 240220-0002.d
Caffeic acid
Counts
x10 6
232.9756
385.1141
611.1372
980.0163
Mass-to-Charge (m/z)

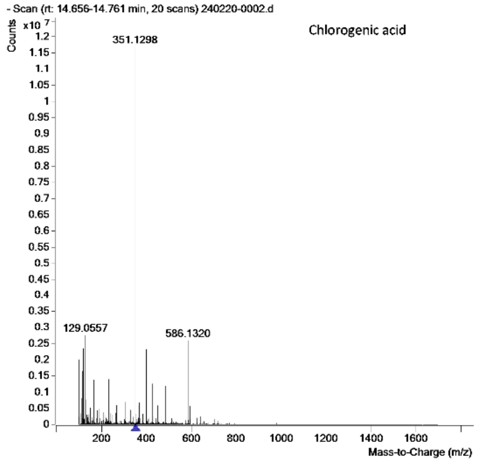

- Scan (rt: 14.656-14.761 min, 20 scans) 240220-0002.d
Chlorogenic acid
Counts
x10 7
351.1298
129.0557
586.1320
Mass-to-Charge (m/z)

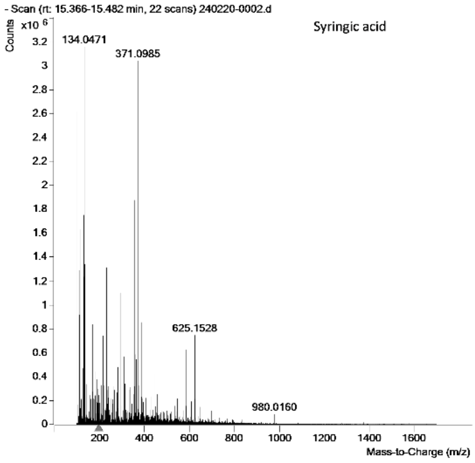

- Scan (rt: 15.366-15.482 min, 22 scans) 240220-0002.d
Syringic acid
Counts
x10 6
134.0471
371.0985
625.1528
980.0160
Mass-to-Charge (m/z)

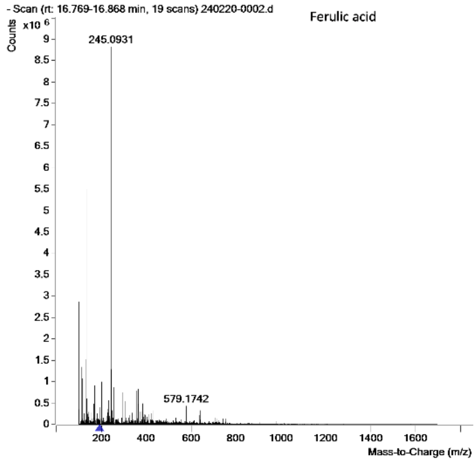

- Scan (rt: 16.769-16.868 min, 19 scans) 240220-0002.d
Ferulic acid
Counts
x10 6
245.0931
579.1742
Mass-to-Charge (m/z)

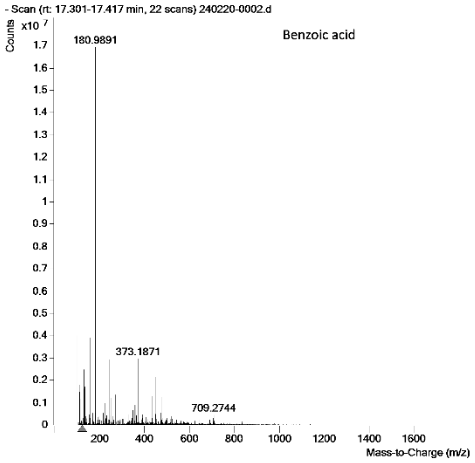

- Scan (rt: 17.301-17.417 min, 22 scans) 240220-0002.d
Benzoic acid
Counts
x10 7
180.9891
373.1871
709.2744
Mass-to-Charge (m/z)
